# Presence of human breast cancer xenograft changes the diurnal profile of amino acids in mice

**DOI:** 10.1038/s41598-022-04994-6

**Published:** 2022-01-19

**Authors:** Rubens Paula Junior, Nathália Martins Sonehara, Bruna Victorasso Jardim-Perassi, Akos Pal, Yasmin Asad, Luiz Gustavo Almeida Chuffa, Roger Chammas, Florence I. Raynaud, Debora A. P. C. Zuccari

**Affiliations:** 1 Faculdade de Medicina de São José Do Rio Preto, São José do Rio Preto, Brazil; 2 Instituto Do Câncer Do Estado de São Paulo, São Paulo, Brazil; 3 The Institute of Cancer Research, London, UK; 4 Universidade Do Estado de São Paulo, São Paulo, Brazil

**Keywords:** Biochemistry, Biological techniques, Cancer

## Abstract

Human xenografts are extremely useful models to study the biology of human cancers and the effects of novel potential therapies. Deregulation of metabolism, including changes in amino acids (AAs), is a common characteristic of many human neoplasms. Plasma AAs undergo daily variations, driven by circadian endogenous and exogenous factors. We compared AAs concentration in triple negative breast cancer MDA-MB-231 cells and MCF10A non-tumorigenic immortalized breast epithelial cells. We also measured plasma AAs in mice bearing xenograft MDA-MB-231 and compared their levels with non-tumor-bearing control animals over 24 h. In vitro studies revealed that most of AAs were significantly different in MDA-MB-231 cells when compared with MCF10A. Plasma concentrations of 15 AAs were higher in cancer cells, two were lower and four were observed to shift across 24 h. In the in vivo setting*,* analysis showed that 12 out of 20 AAs varied significantly between tumor-bearing and non-tumor bearing mice. Noticeably, these metabolites peaked in the dark phase in non-tumor bearing mice, which corresponds to the active time of these animals. Conversely, in tumor-bearing mice, the peak time occurred during the light phase. In the early period of the light phase, these AAs were significantly higher in tumor-bearing animals, yet significantly lower in the middle of the light phase when compared with controls. This pilot study highlights the importance of well controlled experiments in studies involving plasma AAs in human breast cancer xenografts, in addition to emphasizing the need for more precise examination of exometabolomic changes using multiple time points.

## Introduction

Complex diseases such as cancer have been widely studied through omics analysis to understand the mechanism of oncogenesis and to identify new biomarkers^1,2^. Metabolic dysregulation and cell reprogramming is one of the hallmarks of cancer as a direct and indirect consequence of oncogenic mutations^3^. The transforming process from normal to malignant cells is associated with a disturbance in the activity of a number of metabolic pathways. A common feature of cancer cells is the ability to acquire necessary nutrients to maintain viability and build new biomass. The cancer-associated metabolic changes are multiple and include deregulated uptake of various metabolites and use of opportunistic modes of nutrient acquisition^4^. Therefore, to ensure the mechanisms necessary for proliferation, deregulated pro-proliferative and pro-survival signals of cancer cells rewire metabolism to support biosynthesis of proteins, nucleotides, and lipids, as well as production of energy and NADPH^5^.

Studies in cancer cells demonstrated the central role of numerous metabolic pathways in supporting biosynthesis and bioenergetics required for cell growth and proliferation. Metabolomics studies have shown that the levels of many amino acids (AAs) are higher in several types of cancer when compared with the corresponding normal tissue, as a consequence of increasing tumor demand^6^. Characteristics of cancer AAs metabolism networks include (i) elevated consumption of AAs and upregulation of its transporters; (ii) demand for specific non-essential AAs that exceed intracellular supply, leading to dependence on exogenous sources; and (iii) altered enzyme levels that catalyze AAs synthesis and/or catabolism^7^. There is evidence that cancer cells possess the metabolic capability to repurpose their waste molecules, such as lactate from glucose oxidation and ammonia from AAs metabolism, recycling those for its own deregulated metabolism^8,9^.

In all species, most features associated with cellular metabolism including physiological and molecular events, exhibit a diurnal rhythm, which are endogenous and exogenously coordinated by circadian clocks located in central and peripheral tissues as well as by external factors such as light, meals and social timing^10,11^. Metabolic studies, in humans and mice, have shown a range of variation of blood molecules (e.g., amino acids, acylcarnitines, sugars, fatty acids, and phospholipids) following a clock-dependent oscillation^12–14^. Although the intricate connection of the internal circadian clocks, metabolites circulation and cancer remain poorly understood, previous signalling-specific studies have suggested that several aspects of the human plasma metabolites are under rhythmic control^15–17^. The circadian clock coordination may be fundamentally involved in those processes, in the context of cancer, regulating metabolic pathways involved in carbohydrate, amino acid, fatty acid/lipid metabolism^18,19^.

It is noteworthy that an increase in plasma AAs is associated with an increased risk for cancer development and the use of blood-borne tumor-secreted metabolites has been reported as prognostic biomarkers^20,21^. Indeed, circadian analysis of the tumor microenvironment may have a clinically significant value and the time of day may be considered as an important factor to the rhythmic circadian profiles of many metabolites. It should also be mentioned that tumor cells may release their metabolic waste into the bloodstream in a rhythmic manner as many metabolic pathways could be targeted at different peak times of the day. In the present study, we aimed to measure metabolite concentrations throughout 24 h in breast cancer cells (MDA-MB-231) and in non-tumorigenic cells (MCF10A). Moreover, we evaluated in vivo plasma samples of control mice and mice bearing breast cancer (MDA-MB-231) xenograft to verify specific AAs alterations between tumor and non-tumorigenic conditions related to day-time changes at eight zeitgeber time points (ZT).

## Results

### Intracellular metabolites profiling varied in breast cancer cells

The metabolomic profiles of breast cancer cells and normal cells were carried out over 24 h to identify the differences in concentration of intracellular metabolites. In vitro clustering analysis of the metabolites per each class was performed and revealed separation of intracellular metabolites between malignant and normal cells with no effect of time (Figure S1). Amongst six classes of compounds analyzed using the AbsoluteIDQ p180 kit, we observed separated metabolite profiles with the AAs, lyso- and phosphatidylcholine, Biogenic amines and Sphingomyelins.

### AAs are significantly altered in cancer cells across 24 h

**Figure 1:**
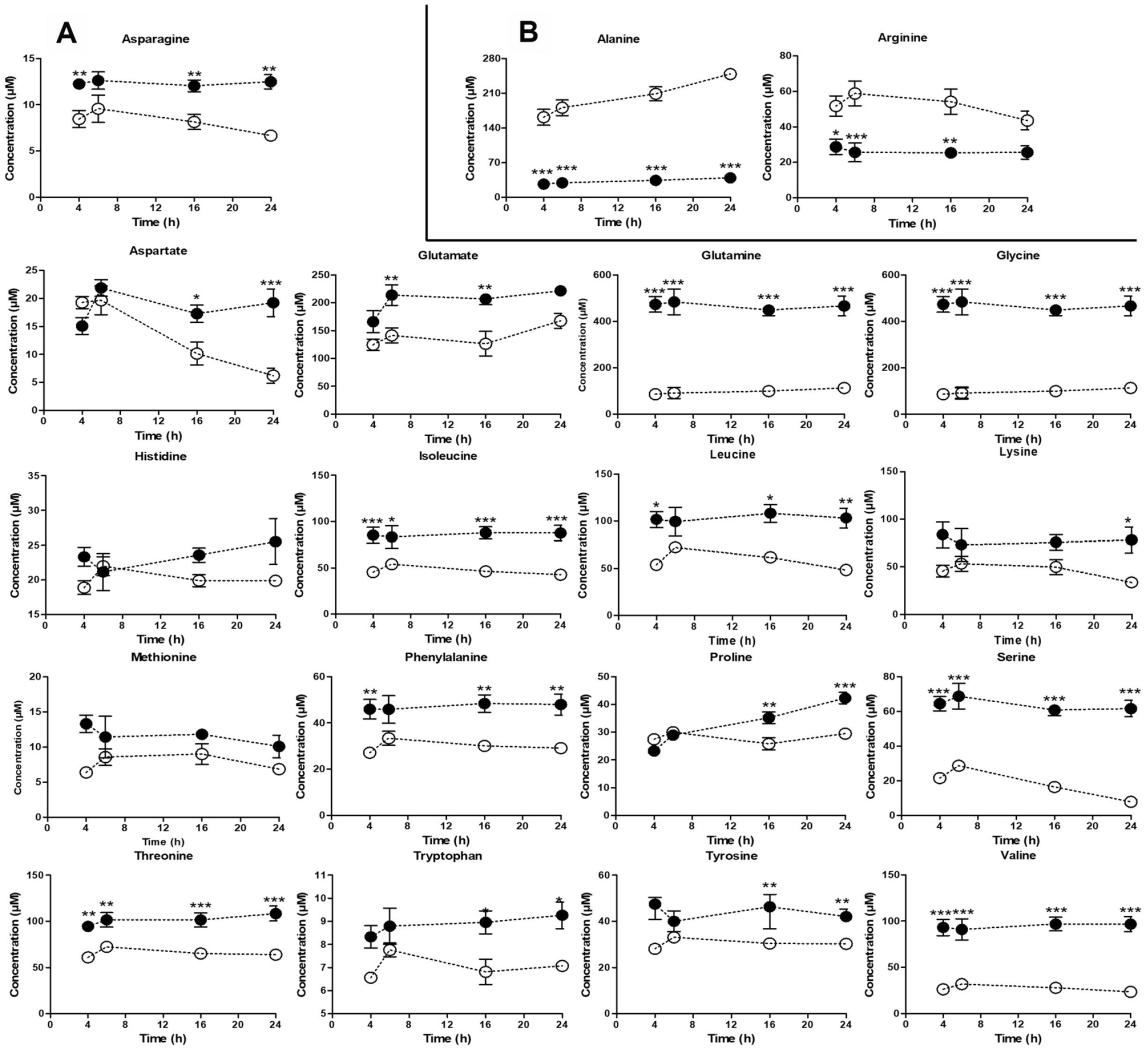
In vitro amino acids concentrations. (**A**) In vitro AAs downregulated in cancer cells (black circles) compared to normal breast cells (white circles). (**B**) In vitro AAs upregulated in cancer cells (black circles) compared to normal breast cells. On the horizontal axis the numbers indicate: four time points (4,6,16 and 24) within 24 h of standard cells growth. The data represent the mean ± S.E.M. of the AAs concentrations comparing MDA-MB-231 cells vs normal human breast cells. *p < 0.01, **p < 0.001, ***p < 0.0001.

21 AAs were potentially detectable in both cell lines; with the exception of Citrulline (**Cit**) where the levels were not consistent and Ornithine (**Orn**) which was undetectable in normal breast cells (data not shown), most of the metabolites were stable and did not vary across 24 h. Whilst two AAs, Alanine (**Ala**) and Arginine (**Arg**), were reduced in MDA-MB-231 cells (*p* < 0.0001), the majority of the AAs including Asparagine (**Asn**), Glutamate (**Glu**), Glutamine (**Gln**), Glycine (**Gly**), Isoleucine (**Ile**), Leucine (**Leu**), Lysine (**Lys**), Methionine (**Met**), Phenylalanine (**Phe**), Serine (**Ser**), Threonine (**Thr**), Tryptophan (**Trp**), Tyrosine (**Tyr**) and Valine (**Val**) were found to be consistently increased (*p* < 0.0001) compared with MCF-10A cells (Fig. 1). At earlier time points, Aspartate (**Asp**) and Proline (**Pro**) levels were lower in cancer cells compared with higher levels in non-tumorigenic cells after 24 h (Fig. 1B). Histidine (**His**) was visibly higher in tumor cells at most time points albeit not significantly. MDA-MB-231 cells are known to have an aggressive phenotype with propensity to metastasis yet they do not have robust circadian rhythms^23^. We used the in vitro data set to analyze the AAs profile per time point. Table 1 shows the analysis of the AAs concentrations between experimental groups in each time point of cell growth (two-way ANOVA followed by Bonferroni’s test). The intracellular AAs profile revealed that only cancer cells had significant alterations (*p* < 0.0001), but day and night time had no significant direct impact on AAs together with the association of length and growth of tumor.

**Table 1:** Differences of amino acids concentrations (pM) in vitro (n = 5) and in vivo (n = 5) between normal and cancer conditions across 24 hours.

Amino acids	In vitro				In vivo			
	Time of sampling (CT)	MCF10A	MDA-MB-231	p value	Time of day (ZT)	Non-tumor bearing mice	Tumor bearing mice	p value
Alanine (Ala) HMDB0000161	4	161.4 ± 32.0	26.6 ± 3.6	p < 0.001	0	646.2 ± 51.2	859.0 ± 114.7	p < 0.05
	6	180.6 ± 31.6	29.2 ± 3.6	p < 0.001	3	561.6 ± 133.5	809.7 ± 102.6	p < 0.05
	16	208.8 ± 28.0	34.3 ± 5.3	p < 0.001	6	853.0 ± 73.1	624.0 ± 96.6	p < 0.01
	24	248.7 ± 15.5	39.4 ± 4.5	p < 0.001	9	435.8 ±118.7	299.6 ± 73.4	N/S
	-	–	–	–	12	633.6 ± 56.7	495.3 ± 94.3	N/S
	–	–	–	–	15	522.0 ± 34.2	563.6 ± 67.5	N/S
	–	–	–	–	18	820.6 ± 58.3	704.3 ± 123.0	N/S
	–	–	–	–	21	774.4 ± 167.2	709.6 ± 114.1	N/S
Arginine (Arg) HMDB0000517	4	51.8 ± 11.4	28.7 ± 8.7	p < 0.05	0	194.7 ± 14.6	267.8 ± 33.9	p < 0.01
	6	58.8 ± 14.2	25.6 ± 10.6	p < 0.001	3	141.0 ± 36.0	287.0 ± 35.1	p < 0.001
	16	54.2 ± 14.2	25.4 ± 4.8	p < 0.01	6	208.3 ± 2.1	135.7 ± 32.1	p < 0.01
	24	43.6 ± 1.6	25.6 ± 7.6	N/S	9	100.6 ± 30.3	49.4 ± 9.4	N/S
	–	–	–	–	12	147.5 ± 7.1	103.2 ± 36.4	N/S
	–	–	–	–	15	97.5 ± 20.6	100.5 ± 17.8	N/S
	–	–	–	–	18	269.7 ± 23.0	203.7 ± 35.1	p < 0.05
	–	–	–	–	21	247.7 ± 10.9	219.8 ± 35.4	N/S
Asparagine (Asn) HMDB0000168	4	8.5 ± 1.8	12.2 ± 1.2	p < 0.01	0	37.9 ± 5.2	49.6 ± 7.7	N/S
	6	9.6 ± 2.1	12.13 ± 1.7	N/S	3	41.5 ± 12.9	61.6 ± 6.3	p < 0.001
	16	8.1 ± 1.6	12.0 ± 1.3	p < 0.01	6	54.9 ± 4.8	39.0 ± 5.1	p < 0.01
	24	6.7 ± 0.5	12.5 ± 1.5	p < 0.001	9	28.3 ± 4.9	25.5 ± 5.9	N/S
	–	–	–	–	12	46.6 ± 5.5	25.2 ± 7.5	p < 0.001
	–	–	–	–	15	31.9 ± 4.4	34.8 ± 4.7	N/S
	–	–	–	–	18	47.9 ± 2.6	34.6 ± 3.3	p < 0.05
	–	–	–	–	21	63.5 ± 7.8	52.5 ± 7.7	N/S
Aspartate (Asp) HMDB0000191	4	19.3 ± 2.2	15.1 ± 3.0	N/S	0	7.1 ± 1.3	10.6 ± 2.8	N/S
	6	19.7 ± 5.1	21.9 ± 3.0	N/S	3	3.8 ± 1.5	10.0 ± 1.9	p < 0.01
	16	10.2 ± 4.1	17.3 ± 3.1	p < 0.05	6	7.5 ± 2.2	12.5 ± 12.6	p < 0.01
	24	6.2 ± 2.7	19.2 ± 5.0	p < 0.001	9	3.7 ± 1.2	5.5 ± 0.2	N/S
	–	–	–	–	12	6.9 ± 2.7	5.5 ± 1.6	N/S
	–	–	–	–	15	4.9 ± 1.6	4.4 ± 2.5	N/S
	–	–	–	–	18	8.3 ± 2.8	7.2 ± 2.0	N/S
	–	–	–	–	21	7.3 ± 1.7	3.7 ± 1.2	N/S
Citrulline (Cit) HMDB0000904	4	–	–	–	0	78.6 ± 8.7	117.8 ± 14.8	p < 0.001
	6	–	–	–	3	73.4 ± 16.9	106.7 ± 18.5	p < 0.01
	16	–	–		6	94.3 ± 5.9	66.4 ± 14.8	p < 0.01
	24	–	–	–	9	57.6 ± 7.2	43.2 ± 10.5	N/S
	–	–	–	–	12	76.5 ± 15.0	54.4 ± 12.0	p < 0.05
	–	–	–	–	15	56.0 ± 7.4	68.9 ± 8.5	N/S
	–	–	–	–	18	115.7 ± 13.3	88.8 ± 0.8	N/S
	–	–	–	–	21	110.2 ± 9.1	94.2 ± 12.4	N/S
Glutamine (Gln) HMDB0000641	4	101.4 ± 32.1	473.5 ± 68.0	p < 0.001	0	876.3 ± 82.0	1029.7 ± 153.7	N/S
	6	90.8 ± 47.9	483.8 ± 111.1	p < 0.001	3	907.6 ± 89.5	998.8 ± 90.3	N/S
	16	99.2 ± 31.6	448.5 ± 47.8	p < 0.001	6	926.3 ± 53.9	788.5 ± 76.3	N/S
	24	112.6 ± 13.4	466.4 ± 86.0	p < 0.001	9	708.0 ± 50.4	787.7 ± 143.3	N/S
	–	–	–	–	12	808.6 ± 24.7	846.2 ± 92.3	N/S
	–	–	–	–	15	861.2 ± 28.0	916.6 ± 55.2	N/S
	–	–	–	–	18	1069.0 ± 76.9	1133.0 ± 163.5	N/S
	–	–	–	–	21	1071.2 ± 157.2	818.3 ± 140.3	p < 0.05
Glutamate (Glu) HMDB0000148	4	124.8 ± 20.1	166.0 ± 39.5	N/S	0	30.8 ± 4.0	33.0 ± 9.2	N/S
	6	141.3 ± 27.1	213.9 ± 36.8	p < 0.01	3	27.0 ± 5.8	24.3 ± 3.7	N/S
	16	126.4 ± 44.2	206.7 ± 19.9	p < 0.01	6	22.8 ± 7.1	44.6 ± 4.7	p < 0.001
	24	167.6 ± 27.0	221.5 ± 8.1	N/S	9	24.7 ± 6.6	42.5 ± 13.7	p < 0.01
	–	–	–	–	12	31.5 ± 2.5	22.2 ± 4.7	N/S
	–	–	–	–	15	26.2 ± 5.1	23.3 ± 7.3	N/S
	–	–	–	–	18	27.4 ± 2.8	44.0 ± 9.6	p < 0.05
	–	–	–	–	21	34.2 ± 7.6	36.7 ± 6.1	N/S
Glycine (Gly) HMDB0000123	4	85.6 ± 8.3	118.6 ± 2.3	p < 0.001	0	333.4 ± 21.2	319.2 ± 50.1	N/S
	6	100.6 ± 47.9	135.1 ± 13.8	p < 0.001	3	283.0 ± 30.0	347.4 ± 24.2	N/S
	16	84.8 ± 31.6	150.6 ± 15.6	p < 0.001	6	386.2 ± 26.1	305.0 ± 53.3	p < 0.05
	24	75.2 ± 13.4	167.6 ± 10.9	p < 0.001	9	266.6 ± 26.4	237.0 ± 48.2	N/S
	–	–	–	–	12	285.8 ± 41.0	234.0 ± 49.	N/S
	–	–	–	–	15	217.2 ± 10.1	263.0 ± 19.0	N/S
	–	–	–	–	18	299.0 ± 38.9	251.2 ± 52.3	N/S
	–	–	–	–	21	319.8 ± 73.8	262.2 ± 48.8	N/S
Histidine (His) HMDB0000177	4	18.9 ± 1.9	21.5 ± 4.1	N/S	0	89.3 ± 11.8	97.7 ± 17.1	N/S
	6	22.0 ± 2.7	21.2 ± 5.3	N/S	3	86.3 ± 9.4	105.3 ± 15.8	N/S
	16	20.7 ± 2.0	22.2 ± 3.2	N/S	6	91.2 ± 7.2	78.5 ± 13.9	N/S
	24	19.9 ± 1.5	22.8 ± 4.1	N/S	9	65.6 ± 10.3	68.0 ± 12.5	N/S
	–	–	–	–	12	84.6 ± 7.3	77.0 ± 11.8	N/S
	–	–	–	–	15	80.1 ± 2.7	90.1 ± 8.5	N/S
	–	–	–	–	18	108.3 ± 8.8	97.3 ± 20.6	N/S
	–	–	–	–	21	111.4 ± 20.6	100.0 ± 14.7	N/S
Isoleucine (Ile) HMDB0000172	4	45.4 ± 5.3	85.3 ± 17.1	p<0.01	0	135.0 ± 17.4	183.0 ± 6.1	P<0.01
	6	54.1 ± 8.7	83.4 ± 24.4	P < 0.05	3	111.0 ± 32.2	174.3 ± 19.4	P<0.001
	16	46.3 ± 5.1	87.8 ± 13.1	P<0.001	6	127.7 ± 5.5	86.2 ± 29.1	N/S
	24	42.6 ± 3.3	87.6 ± 16.7	P<0.001	9	105.6 ± 15.5	104.4 ± 10.0	N/S
	–	–	–	–	12	177.7 ± 48.0	115.9 ± 26.1	p<0.001
	–	–	–	–	15	109.0 ± 11.4	114.3 ± 14.4	N/S
	–	–	–	–	18	178.2 ± 10.1	135.0 ± 11.8	P < 0.05
	–	–	–	–	21	218.3 ± 6.5	170.2 ± 25.7	p<0.01
Leucine (Leu) HMDB0000687	4	63.6 ± 17.6	101.8 ± 17.0	P < 0.05	0	200.6 ± 17.7	297.7 ± 17.2	p<0.01
	6	72.2 ± 19.2	99.4 ± 30.7	N/S	3	147.5 ± 46.0	261.7 ± 28.3	p<0.001
	16	61.6 ± 20.4	108.2 ± 18.8	P < 0.05	6	208.0 ± 4.0	137.1 ± 53.1	N/S
	24	48.2 ± 12.1	103.1 ± 21.1	p<0.01	9	146.2 ± 26.9	147.0 ± 10.8	N/S
	–	–	–	–	12	250.2 ± 59.0	153.7 ± 29.2	p<0.01
	–	–	–	–	15	153.0 ± 15.6	168.8 ± 9.9	N/S
	–	–	–	–	18	290.5 ± 18.6	213.7 ± 12.9	P < 0.05
	–	–	–	–	21	276.4 ± 63.9	257.0 ± 36.5	N/S
Lysine (Lys) HMDB0000182	4	45.6 ± 12.4	83.7 ± 27.3	N/S	0	506.2 ± 22.5	694.0 ± 71.7	p<0.01
	6	53.4 ± 15.8	73.0 ± 34.5	N/S	3	470.0 ± 69.0	709.0 ± 69.8	p<0.001
	16	49.9 ± 15.7	75.6 ± 16.5	N/S	6	610.6 ± 34.5	384.6 ± 92.	p<0.001
	24	33.6 ± 9.8	78.1 ± 27.0	p<0.05	9	266.4 ± 63.9	224.6 ± 42.9	N/S
	–	–	–	–	12	369.0 ± 11.5	258.7 ± 90.0	N/S
	–	–	–	–	15	269.6 ± 26.6	305.8 ± 43.2	N/S
	–	–	–	–	18	520.4 ± 61.3	368.0 ± 90.4	p<0.01
	–	–	–	–	21	553.0 ± 85.9	492.0 ± 42.2	N/S
Methionine (Met) HMDB0000696	4	8.3 ± 2.2	13.3 ± 2.1	N/S	0	72.7 ± 8.9	106.3 ± 6.6	P < 0.05
	6	7.7 ± 2.0	11.4 ± 4.2	N/S	3	69.2 ± 20.0	115.7 ± 21.2	p<0.001
	16	9.0 ± 2.5	11.8 ± 1.2	N/S	6	79.1 ± 6.9	47.5 ± 8.0	P < 0.05
	24	7.6 ± 1.7	10.1 ± 3.2	N/S	9	38.1 ± 5.1	27.0 ± 5.9	N/S
	–	–	–	–	12	54.4 ± 5.7	38.0 ± 5.4	N/S
	–	–	–	–	15	51.4 ± 12.4	48.2 ± 6.7	N/S
	–	–	–	–	18	120.7 ± 7.5	99.3 ± 11.1	N/S
	–	–	–	–	21	102.6 ± 16.7	85.4 ± 32.9	N/S
Ornithine (Orn) HMDB0000214	4	–	–	–	0	101.5 ± 17.5	159.0 ± 17.6	p<0.01
	6	–	–	–	3	96.4 ± 26.5	171.0 ± 23.6	p<0.001
	16	–	–	–	6	91.6 ± 9.0	53.3 ± 17.0	P < 0.05
	24	–	–	–	9	50.0 ± 11.2	37.4 ± 8.0	N/S
	–	–	–	–	12	98.9 ± 15.4	52.3 ± 23.8	P < 0.05
	–	–	–	–	15	66.8 ± 15.8	78.8 ± 21.2	N/S
	–	–	–	–	18	162.4 ± 20.3	90.7 ± 11.1	p<0.001
	–	–	–	–	21	158.6 ± 31.9	152.4 ± 23.9	N/S
Phenylalanine (Phe) HMDB0000159	4	27.1 ± 2.8	45.9 ± 8.4	p<0.01	0	143.0 ± 9.2	184.5 ± 25.6	P < 0.05
	6	33.4 ± 6.1	45.9 ± 11.9	N/S	3	126.0 ± 30.7	183.7 ± 7.1	p<0.01
	16	30.0 ± 3.0	48.4 ± 7.5	p<0.01	6	160.7 ± 1.5	114.8 ± 30.9	P < 0.05
	24	29.1 ± 2.0	48.0 ± 9.1	p<0.01	9	112.0 ± 4.5	95.0 ± 4.6	N/S
	–	–	–	–	12	175.7 ± 17.0	133.7 ± 28.0	P < 0.05
	–	–	–	–	15	126.0 ± 10.2	132.0 ± 13.4	N/S
	–	–	–	–	18	194.8 ± 11.7	154.0 ± 20.2	P < 0.05
	–	–	–	–	21	178.6 ± 20.6	174.0 ± 20.5	N/S
Proline (Pro) HMDB0000162	4	28.1 ± 1.5	23.0 ± 1.3	N/S	0	146.0 ± 18.3	208.0 ± 30.4	p<0.01
	6	30.0 ± 1.7	28.9 ± 2.1	N/S	3	84.8 ± 28.5	135.5 ± 25.3	P < 0.05
	16	25.8 ± 4.4	35.2 ± 4.1	p<0.01	6	175.6 ± 11.1	117.1 ± 28.1	p<0.001
	24	29.5 ± 1.2	42.3 ± 4.1	p<0.001	9	62.0 ± 8.4	44.4 ± 12.2	N/S
	–	–	–	–	12	161.0 ± 39.3	107.2 ± 4.0	p<0.01
	–	–	–	–	15	69.7 ± 10.9 ±	80.2 ± 10.4	N/S
	–	–	–	–	18	210.4 ± 19.5	150.3 ± 19.3	p<0.01
	–	–	–	–	21	150.0 ± 29.6	116.3 ± 19.1	N/S
Serine (Ser) HMDB0000187	4	21.6 ± 1.4	64.4 ± 8.2	p<0.001	0	170.3 ± 7.1	206.7 ± 22.5	N/S
	6	28.7 ± 4.9	68.7 ± 14.7	p<0.001	3	159.5 ± 22.5	215.4 ± 13.5	p<0.01
	16	16.4 ± 2.0	60.7 ± 6.3	p<0.001	6	232.8 ± 23.8	160.4 ± 35.5	p<0.001
	24	7.9 ± 1.1	61.6 ± 9.4	p<0.001	9	115.8 ± 18.1	93.3 ± 23.3	N/S
	–	–	–	–	12	159.8 ± 9.4	101.7 ± 24.7	p<0.01
	–	–	–	–	15	126.8 ± 13.8	135.6 ± 14.7	N/S
	–	–	–	–	18	201.3 ± 11.0	155.8 ± 25.8	P < 0.05
	–	–	–	–	21	220.5 ± 24.1	179.2 ± 32.1	N/S
Threonine (Thr) HMDB0000167	4	61.1 ± 4.9	94.5 ± 11.5	p<0.01	0	175.4 ± 21.3	190.8 ± 40.9	N/S
	6	72.4 ± 7.8	101.7 ± 15.5	p<0.01	3	176.6 ± 42.5	202.0 ± 36.9	N/S
	16	65.2 ± 4.7	101.5 ± 15.3	p<0.001	6	173.4 ± 16.7	129.9 ± 26.9	N/S
	24	64.0 ± 1.8	108.4 ± 16.2	p<0.001	9	114.5 ± 17.9	85.4 ± 20.4	N/S
	–	–	–	–	12	138.8 ± 19.5	110.3 ± 24.2	N/S
	–	–	–	–	15	138.8 ± 19.5	147.8 ± 18.2	N/S
	–	–	–	–	18	226.0 ± 12.6	197.8 ± 62.5	N/S
	–	–	–	–	21	238.0 ± 30.9	223.4 ± 22.1	N/S
Tryptophan (Trp) HMDB0000929	4	6.6 ± 0.4	8.3 ± 1.0	N/S	0	116.8 ± 20.9	132.4 ± 15.9	N/S
	6	7.8 ± 0.6	8.8 ± 1.6	N/S	3	111.5 ± 32.7	123.6 ± 25.7	N/S
	16	6.8 ± 1.1	9.0 ± 1.0	P < 0.05	6	111.2 ± 20.1	101.5 ± 21.8	N/S
	24	7.1 ± 0.5	9.3 ± 1.2	P < 0.05	9	98.4 ± 10.3	121.1 ± 34.9	N/S
	–	–	–	–	12	130.8 ± 26.2	129.2 ± 30.9	N/S
	–	–	–	–	15	76.6 ± 7.8	70.7 ± 7.2	N/S
	–	–	–	–	18	132.0 ± 13.7	129.2 ± 36.6	N/S
	–	–	–	–	21	126.2 ± 23.5	115.2 ± 19.4	N/S
Tyrosine (Tyr) HMDB0000158	4	28.1 ± 2.9	42.8 ± 7.9	p<0.01	0	143.0 ± 16.8	174.0 ± 46.7	N/S
	6	33.1 ± 4.5	38.0 ± 5.7	N/S	3	131.9 ± 28.0	149.4 ± 15.2	N/S
	16	30.4 ± 3.1	45.3 ± 6.5	p<0.01	6	139.8 ± 25.9	102.2 ± 17.3	p<0.001
	24	30.3 ± – 2.0	43.0 ± 2.0	p<0.01	9	135.5 ± 45.1	65.7 ± 14.9	N/S
	–	–	–	–	12	175.4 ± 18.7	86.9 ± 30.7	N/S
	–	–	–	–	15	78.6 ± 11.6	72.0 ± 7.9	N/S
	–	–	–	–	18	96.2 ± 22.9	131.4 ± 45.6	N/S
	–	–	–	–	21	72.0 ± 10.0	117.1 ± 17.7	N/S
Valine (Val) HMDB0000883	4	26.3 ± 2.7	93.1 ± 17.6	p<0.001	0	263.3 ± 32.7	313.7 ± 18.0	N/S
	6	32.0 ± 4.9	91.0 ± 22.9	p<0.001	3	217.0 ± 64.5	343.3 ± 23.4	P < 0.05
	16	27.9 ± 2.2	96.8 ± 14.7	p<0.001	6	267.7 ± 16.3	189.7 ± 56.3	N/S
	24	23.6 ± 2.2	96.7 ± 16.2	p<0.001	9	228.2 ± 27.0	213.8 ± 23.0	p<0.001
	–	–	–	–	12	311.0 ± 37.9	251.8 ± 43.0	P < 0.05
	–	–	–	–	15	267.0 ± 13.3	233.3 ± 17.6	N/S
	–	–	–	–	18	354.4 ± 16.8	285.7 ± 28.5	N/S
	–	–	–	–	21	433.0 ± 13.1	329.5 ± 71.4	N/S

All values are mean ± SD; *p*-values are from Two-way analysis of variance.

*CT = culture time; ZT (zeitgeber).

### Plasma AAs are affected by time of day and presence of a tumor

**Figure 2:**
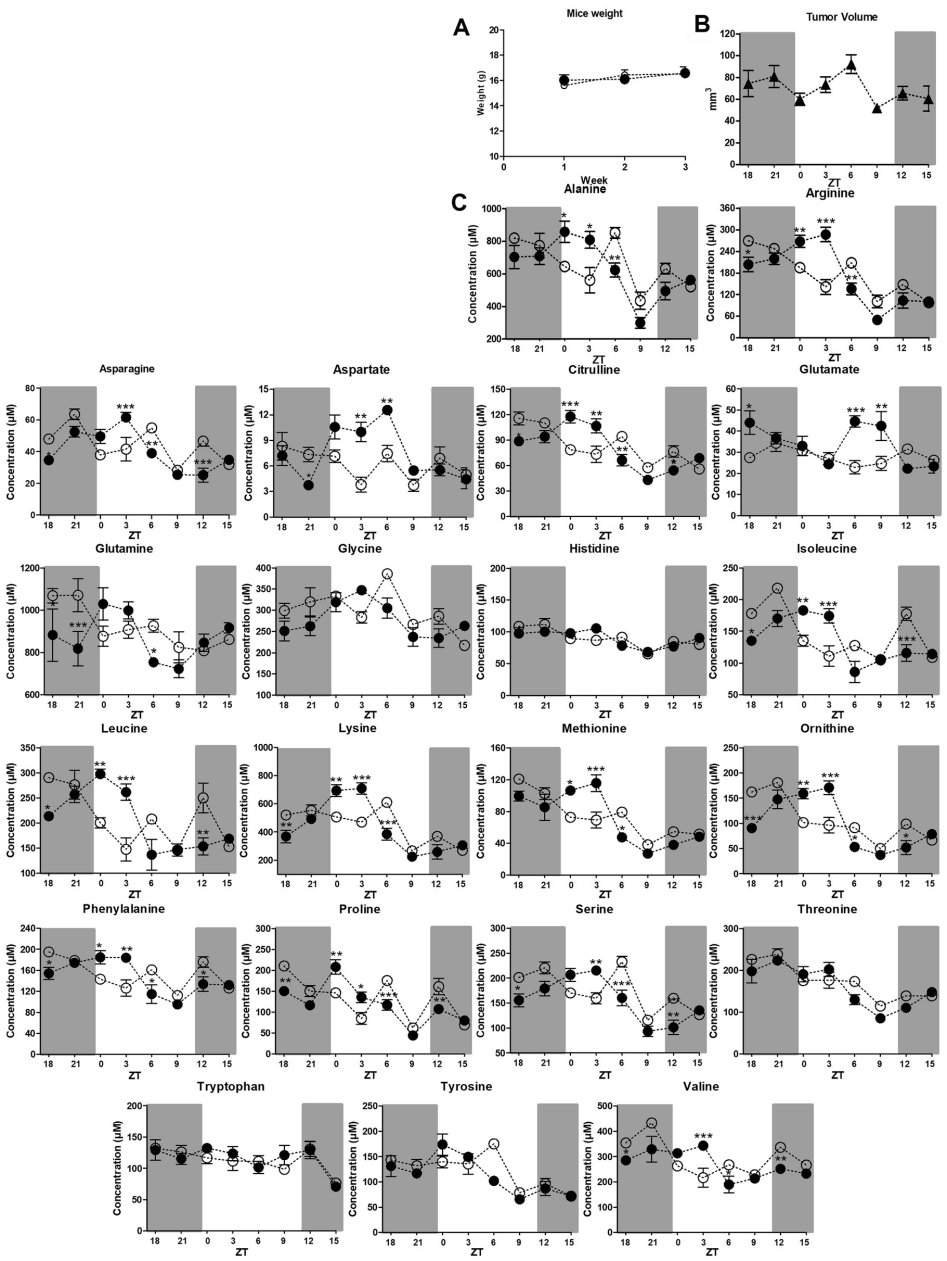
Body weight, tumor volume and plasma amino acids concentrations in mice. (**A**) Mice weight represented by the mean ± S.E.M across three weeks of tumor hosting. (**B**) Tumor volume represented by the mean ± S.E.M in each time point when mice were bled. (**C**) In vivo AAs detected in tumor-bearing animals (black circles) compared to control (white circles). On the horizontal axis the numbers indicate eight time points, Zeitgeber time (ZT), across a normal diurnal variation with 12 h in light phase (clear background) and 12 h in dark phase (dark background), starting at ZT18 and finishing at ZT15. The data represent the mean ± S.E.M. comparing tumor-bearing animals vs non-tumor-bearing animals. *p < 0.01, **p < 0.001, ***p < 0.0001.

Following in vivo experiments, plasma samples were taken since they are a reliable source of blood biomarkers. We observed no changes in the body weight of the mice over the 22 days of tumor-hosting (Fig. 2A). The tumor volumes varied from 50 to 98 mm^3^ and the tumor tissue did not show necrotic regions post excision (Fig. 2B).

Evaluation of the concentrations of all plasma metabolites in the eight time points revealed that AAs were the only metabolite class significantly altered between control and tumour bearing mice. (Fig. 1) Analysis of variance showed that in vivo*,* plasma AAs levels in mice were significantly affected not only by tumor presence (*p* < 0.0015) and diurnal variation (*p* < 0.0001), but also by the time/tumor interaction (*p* < 0.0001).

In non-tumor bearing mice, we detected significant higher levels of most of the AAs in the late dark phase including **Arg** (*p* < 0.05); **Asn** (*p* < 0.05); **Asp** (*p* < 0.05); **Cit** (*p* < 0.05); **Gln** (*p* < 0.05; *p* < 0.001); **Ile** (*p* < 0.05; *p* < 0.01); **Leu** (*p* < 0.05); **Orn** (*p* < 0.001); **Phe** (*p* < 0.05); **Pro** (*p* < 0.01); **Ser** (*p* < 0.05) and **Val** (*p* < 0.05) when compared with tumor-bearing mice in which lower plasma levels were detected in late dark phase. In early light phase, AAs were lower in the plasma of control animals compared with tumor-bearing mice which had significantly higher plasma concentrations of **Ala** (*p* < 0.05; *p* < 0.01); **Arg** (*p* < 0.01; *p* < 0.001); **Asn** (*p* < 0.001); **Asp** (*p* < 0.05); **Gln** (*p* < 0.05); **Ile** and **Leu** (*p* < 0.01; *p* < 0.001); **Lys** (*p* < 0.01; *p* < 0.001); **Met** (*p* < 0.05; *p* < 0.001); **Phe** (*p* < 0.05; *p* < 0.01); **Pro** (*p* < 0.01; *p* < 0.05); **Ser** (*p* < 0.01); **Val** (*p* < 0.001) (Fig. 2C).

In contrast to that observed early in the day, the middle of the light phase showed significantly lower plasma AAs levels in tumor-bearing mice compared with control mice. These include **Ala** (*p* < 0.01); **Arg** (*p* < 0.01); **Asn** (*p* < 0.01); **Cit** (*p* < 0.01); **Lys** (*p* < 0.001); **Met** (*p* < 0.05); **Orn** (*p* < 0.05); **Phe** (*p* < 0.05); **Pro** (*p* < 0.001); **Ser** (*p* < 0.001); **Val** (*p* < 0.05). In early dark phase, we observed a number of AAs slightly decreased in tumor-bearing mice, whereas healthy mice showed significant increase of **Asn** (*p* < 0.001); **Cit** (*p* < 0.05); **Ile** (*p* < 0.001); **Leu** (*p* < 0.01); **Orn** (*p* < 0.05); **Phe** (*p* < 0.05); **Pro** (*p* < 0.01); **Ser** (*p* < 0.01); **Val** (*p* < 0.01). At ZT15, the plasma levels of AA in both groups were similar with no statistical significance (Fig. 2C). The overall observed C_max_ of Arg, Asp, Lys, Met, and Orn was significantly higher in plasma of tumor-bearing mice compared with control animals. The C_max_ occurred at different times in both tumor bearing mice and non-tumor bearing mice and the C_min_ occurred at the same time.

**Figure 3:**
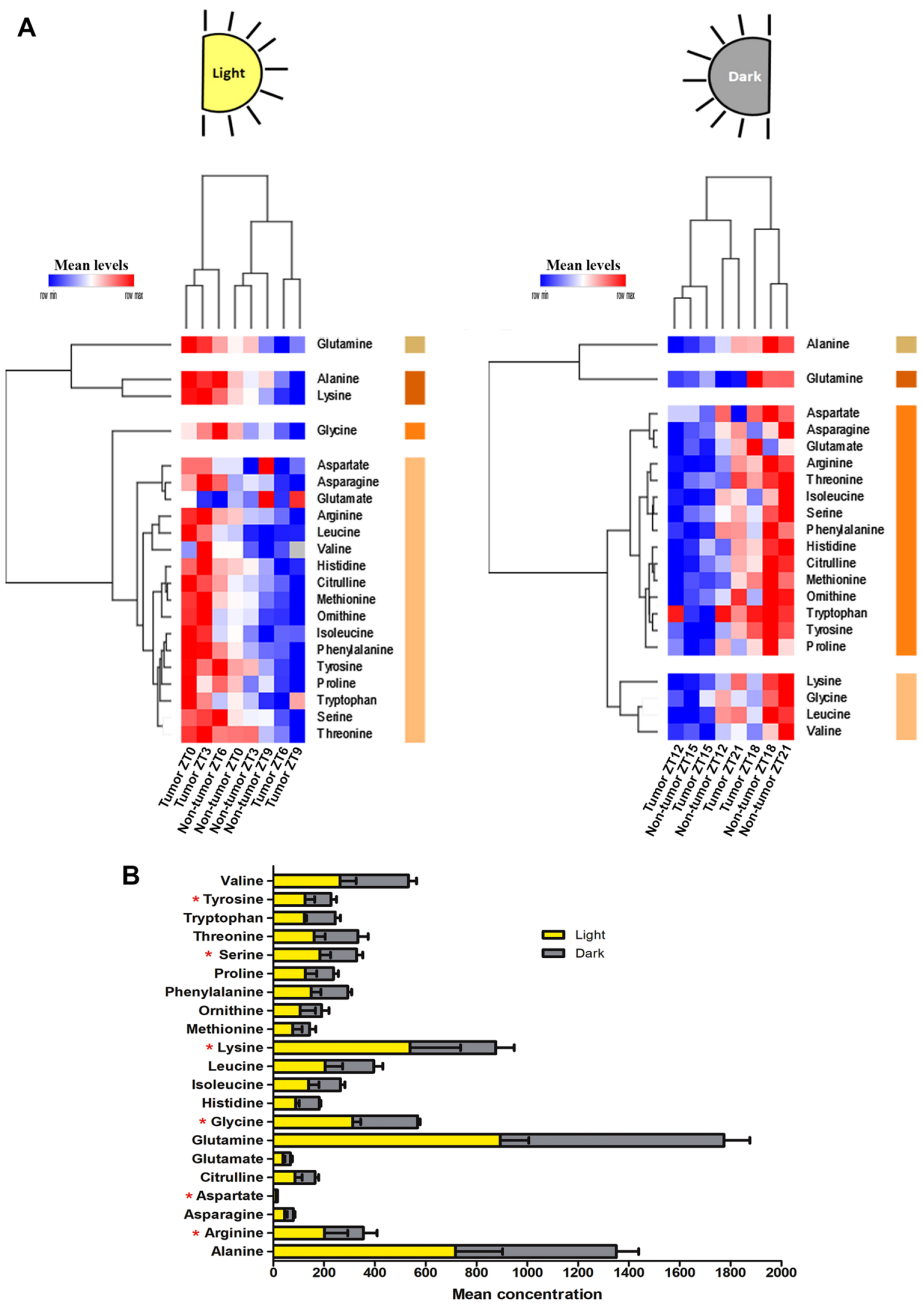
Profile of AAs levels evaluated daily in tumor-bearing and non-tumor bearing mice. (**A**) Heat maps illustrating the normalized mean levels of AAs, visualized as minimum “blue”-maximum “red” values. We used two factors of clusterization (AAs by row and tumor presence by column; Euclidean distance analysis). The right-aligned colors indicate the four main clusters of AAs in the light and dark phases. Light phase included ZT0, ZT3, ZT6 and ZT9 periods. Dark phase included ZT12, ZT15, ZT18 and ZT21 periods**.** (**B**) Horizontal bar graphic showing the differences in plasma AAs (mean concentration ± S.E.M.) between light (ZT0, 3, 6, and 9) and dark (ZT12, 15, 18, and 21) phases in the presence of tumor. *P < 0.05.

Although clustering analyses clearly separated the profile of AAs between tumor-bearing and non-tumor bearing mice across 24 h, a noticeable reduction in plasma AAs levels by tumor-bearing animals occurred during advanced light and dark phases (Fig. 3). During the day, we identified four main clusters of AAs varying among the groups (**Gln**, **Ala-Lys, Gly,** and other AAs), whereas in the dark phase the main distinct clusters were **Gln, Ala**, **Lys-Gly-Leu-Val**, and other AAs (Fig. 3 A). Whilst most AAs were significantly increased in tumor bearing animals during the initial light phase, a significant reduction of AAs occurred during dark phases. Examination of AAs concentrations throughout the day and night, showed a different metabolite profile pattern in the presence of tumor. A significant reduction of plasma AAs was observed in the dark phase, for the following AAs (**Arg**, **Asp**, **Lys**, **Gly**, **Ser**, and **Tyr**) (Fig. 3B).

### Enrichment analysis for altered metabolic pathways

**Figure 4:**
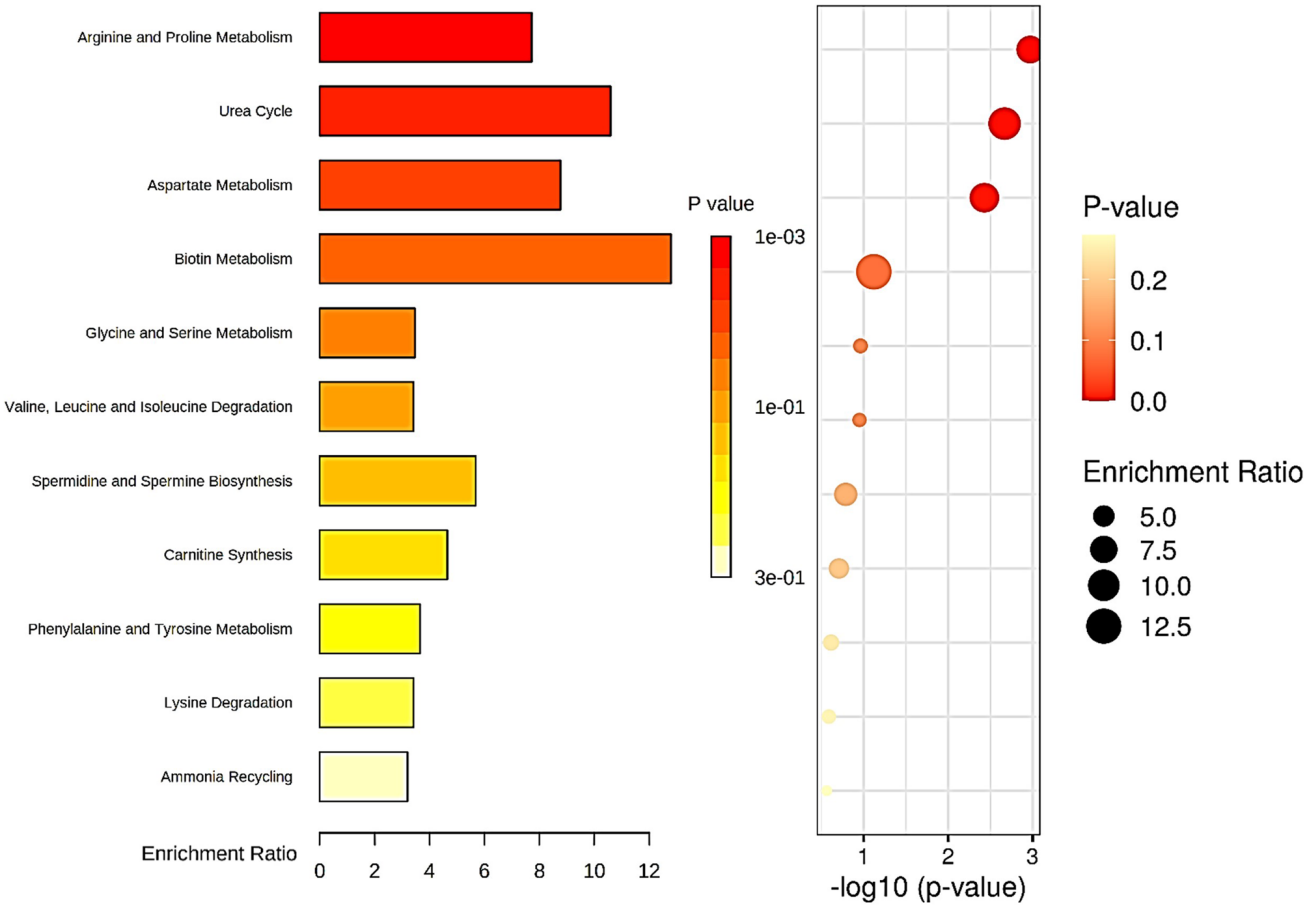
Top enrichment analysis for Arg, Asn, Cit, Ile, Leu, Lys, Orn, Phe, Pro, and Ser. Analysis was performed in Metaboanalyst with those AAs listed as metabolites within the Human Metabolome Database HMDB and showed significant alteration in 11 pathways in triple negative MD-MBA-231 breast cancer xenograft model. The three most significant biological pathways enriched include Arg and Pro metabolism (*p* = 0.00107); Urea Cycle (*p* = 0,00,215) and Asp metabolism (*p* = 0.00373) followed by Biotin metabolism (*p* = 0.0758); Gly and Ser metabolism (*p* = 0.109); Val, Leu and Ile degradation (*p* = 0.112); Spermidine and Spermine biosynthesis (*p* = 0.163); Carnitine synthesis (*p* = 0.196); Phe and Tyr metabolism (*p* = 0.243); Lys degradation (*p* = 0.258); Ammonia recycling (*p* = 0.273).

The enrichment analysis was based on AAs profile depicting at least four time points with significant differences between the in vitro experimental groups and the shifting plasma profiles in the early light phase. Thus, the enrichment analysis was performed with **Arg**, **Asn**, **Cit**, **Ile**, **Leu**, **Lys**, **Orn**, **Phe**, **Pro,** and **Ser,** and displayed 11 human metabolic pathways in which those AAs are significantly involved. The highest significant involvement and most enriched pathways **Arg**, **Pro** and **Asp** metabolism plus Urea Cycle was achieved (*p* < 0.005) although **Asp** was not included the 10 AAs selected. Other biological pathways associated with the AAs were enriched (*p* < 0.3) including Biotin, **Gly** and **Ser** metabolism again despite **Ser** not amongst the AAs chosen. Most of the enriched pathways are linked to several biological processes including AAs amino acids catabolism, cell growth, proliferation, migration, invasion, metastasis and synthesis of biomolecules (Fig. 4).

## Discussion

Our study shows that mice bearing human breast cancer MDA-MB-231 xenografts exhibit broad differences in the rhythm of plasma AAs compared with the non-tumor bearing mice. The C_max_ of most AAs is observed in the light phase before mid-day in tumor bearing mice in contrast to that occurring in the middle of the dark phase in control animals. There is no real difference in C_max_ yet the C_min_ is decreased in tumor bearing mice where it occurs at the same time as the control mice. In this study, we emphasized that measurement of plasma AAs levels at a single time could lead to opposite results depending on time-sampling.

We reported a clear metabolite separation between malignant and normal conditions in vitro, across all time points evaluated. There were no rhythmic variations in metabolites between normal and cancer cells over the four time points across 24 h. Meanwhile, in vivo, metabolites were clustered according to time and at the opposite corresponding time points. In our xenograft model, we did not observe any change in the body masses of mice hosting the tumor. In these animals, specific rhythmic AAs variation was clearly observed between the groups and during the analysis with eight time points across 24 h (Fig. 2). In tumor-bearing mice, we observed lower levels of selected AAs in late dark phase which tended to increase until the early light phase; in contrast to healthy mice where AAs levels were higher in late dark phase and decreased plasma levels in early light phase. Tumor-bearing mice showed peak levels of more than half of the AAs with a significant shifting profile of those selected AAs in early light phase (ZT0 and ZT3). However, in late light phase (ZT6), all plasma AAs levels of tumor-bearing mice, excluding aspartate, were decreased compared with healthy mice. At ZT9, the plasma levels of selected AAs decreased and showed no significant difference between the groups. In early dark phase (ZT12), tumor-bearing mice had significantly lower plasma levels of selected AAs compared with control mice. Interestingly, at ZT15, all the AAs showed similar concentrations with no significance in both experimental groups.

None the less, the other metabolite classes analyzed showed two or one time point(s) with significant differences between the groups and yet did not show the similar profile alteration between the groups, observed with the AAs. In the current study, food was provided ad libitum and the likelihood of animals feeding at different times is unlikely. Should this be the reason for our observed differences? If various feeding times had affected the metabolic profiles, one would also expect to see parallel differences on other classes of metabolites (e.g., carnitines) which was not observed suggesting that variation in food intake is not responsible for the changes observed.

The reason for the current observation is not clear at all. Different clocks are present in different tissues and it has recently been shown that metabolism in tumor cells is driven primarily by the local tissue metabolism^25^. Of note, the gene expression rhythm profile in the liver of mice bearing the 4T1 breast cancer xenograft was modified and a subset of clock genes showed a secondary peak when compared with control animals^26^. In this study, there was no evidence that tissue or circulating amino acids were altered. However, in another study with genetically engineered mice, the development of lung adenocarcinoma driven by conditional activation of the KrasG12D mutation and loss of the p53 tumor suppressor led to altered circadian patterns of transcription of a subset of genes in the liver^27^. This phenomenon was observed without alterations to the core circadian clock machinery. These transcriptional changes resulted in altered patterns of liver metabolism across the circadian cycle, including insulin signaling, glucose production, and lipid metabolism. None of these studies evaluated the circulating metabolites. It is therefore possible that the presence of a breast cancer MDA-MB-231 cells-originated tumor affects liver metabolism resulting in altered plasma AAs; this needs further validation^26^.

Changes in AAs metabolism and concentration in cancer patients have been consistently investigated^20,28^. A study with plasma AAs reported decreased levels in pancreatic and breast cancers and these levels were correlated with an enhanced consumption of AAs by the tumor^20^. Furthermore, several AAs have been reported with lower levels in patients with breast, gastric, and thyroid cancer^29,30^. Conversely, plasma AAs levels have been shown to be increased in a number of studies considering the early stages of cancer. In addition, plasma AAs levels have been shown to be tumor-dependent and to correlate with specific breast cancer subtypes^21,31,32^. Previous investigation focusing on breast cancer using a metabolomics approach showed that AAs metabolism represent some of the changes in metabolic activity of several pathways associated with breast cancer^33^. It also should be noted that cellular uptake of AAs correlates with cancer cell proliferation as their exogenous supply may critically affect the proliferative activity, survival or tumorigenic potential of cancer cells^34^.

Indeed, solid tumors provide a nutrient-poor environment where cells often outgrow their small molecules from the host blood supply. It is possible that cancer metabolism affects the circulating AAs levels and its availability may be clock-controlled^17–19^. The enrichment analysis shows major significant association with three metabolic pathways such as Arginine and Proline, Glycine and Serine metabolism, and Urea Cycle, all involved with molecular biosynthesis and degradation. In addition to the Warburg effect, metabolism of AAs, i.e. glutamine, serine, aspartate, and proline, has been shown to contribute to tumor metabolic reprogramming^35–37^. Altered levels of Arg plus Orn and Cit may have affected the urea cycle in our animal model. This pathway has a plethora of important self-maintained enzymatic steps such as argininosuccinate synthetase 1 (ASS1) and ornithine transcarbamylase (OTC). These enzymes have been reported in humans and mice, displaying clock-dependent activity in a rhythmic manner, which contributes to the temporal plasma variation and biosynthesis of these AAs^17^. Circadian rhythm dependency has also been documented regarding homocysteine levels as being an enzyme component cyclically coordinated by the clock genes^37^.

De novo Pro, Ser and Gly synthesis has been shown to be critical in several types of cancer^37,38^. Pro catabolism is reported in in vivo metastasis formation through high expression of proline dehydrogenase (PRODH) compared to primary breast cancers of patients and mice. In fact, Pro can be interconvertible with Glu, in which glutamic-c-semialdehyde (GSA) is utilized as an intermediate, which is derived from Glu or Pro and converted into Orn, serving as a precursor for Arg synthesis in the Urea cycle^39^. Meanwhile, Ser metabolism may be an important factor for breast cancer as Ser biosynthetic pathways were upregulated in high metastatic breast cancer^40^. Glycine metabolism is also involved in cancer cell proliferation being able to promote tumorigenesis and malignancy where it provides carbon and nitrogen for energy^41^. Research into plasma AAs levels started decades ago with Bennegård et al. (1984) which examined 18 cancer patients with more than 7% weight loss and found a significant decrease in Pro and Ser levels^42^. Watanabe et al. (1984) have also investigated PFAA concentrations in 14 cirrhotic patients with hepatocellular carcinoma (HCC) and found significant decreases in the levels of Ser^43^. A well described phenomenon that affects plasma amino acid in cancer is cachexia. Plasma levels of AAs have been shown to increase or decrease in various studies undergoing significant muscle loss^44^. These differences could potentially be due to sampling time collection but also to the fact that it is difficult to trace the source of effects in plasma (e.g., the tumor or a peripheral effect such as cachexia or liver metabolism alteration). In our in vivo study, the tumors were relatively small and no body weight loss was observed suggesting no associated cachexia.

In our study, there was no sign of rhythmic intracellular concentrations in MD-MBA-231 and non-tumorigenic mammary cells within 24 h of standard cell culture. Conversely, the plasma AAs in in vivo experiments showed an evident and significant cyclical behaviour during diurnal variation in mice. Thus, AAs were observed being increased or decreased depending on daytime. The lack of rhythmic variation in our in vitro study is likely due to the cells having their own biological clocks or the tissue culture conditions (different from in vivo xenografts), which are peripheral and non-synchronized within any central circadian oscillator. The mice might have their circulating metabolites under their own controlling central oscillator. Once in the xenograft model, the cells could have resynchronized their clock according to the new host’s central oscillator^45^. We suggest that depending on the time we may verify an increased or a decreased level which may be a general tumor association with the diurnal effect.

There are no currently reported data supporting a circadian control of Pro and Asp metabolism and the enriched pathways. Most of the pathways such as Met, Tyr, Phe and Biotin metabolism, Val, Leu, Ile and Lys degradation, carnitines synthesis, ammonia recycling are subjected to metabolic reprogramming to sustain cell growth, proliferation, and oncogenes expression in many cancer types; these are essential to accumulate building blocks for the construction of new cellular components^46–49^.

Tumors also can affect circadian rhythms even in tumor-free organs. For example, mice bearing triple negative breast cancer can impact the hepatic circadian gene expression causing alterations in several genes including the core clock genes Rev-Erba (Nr1d1), PER2, RORγ, and CLOCK^26^. Dysregulation of these physiological oscillations can further result in oxidative stress, polyploidy, and inflammation. The presence of a non-metastatic melanoma significantly impaired the biological clock of tumor-adjacent skin and affected the oscillatory expression of genes involved in light- and thermo-reception, proliferation, melanogenesis, and DNA repair. The expression of tumor molecular clock was significantly reduced compared to healthy skin but still displayed an oscillatory profile (attenuated PER1 and BMAL1 oscillations)^50,51^. Although not affecting the core clock machinery, Masri et al. also found that lung cancer induced significant shifts in liver circadian rhythms, both at the level of transcripts and metabolites including AAs in tumor-bearing mice. Yet, nutrient addiction of a tumor subtype can differ. For instance, when glutamine levels are limiting, cell proliferation can be alternatively driven by aspartate or asparagine^52^. This establishes an interesting paradox whereby limiting the availably of a specific nutrient may not inhibit cancer cell proliferation, as tumors can switch their fuel preference. It should be emphasized that plenty of these nutrients are rhythmically offered over the day, thus it may be possible that fuel utilization of tumors may differ based on rhythmic availability of nutrients^53–55^.

The recent crosstalk between the gut microbiota and cancer emerged to directly and/or indirectly correlate with tumor development, treatment, and prognosis^56–58^. Disruptions to gut microbiota balance or “dysbiosis” is implicated in a growing list of cancers and biological processes including host cell proliferation and apoptosis, immune system function, chronic inflammation, oncogenic signaling, hormonal, and detoxification pathways^57,58^. Most of the studies report the main effects of gut microbiota on cancer development correlating with the inflammation caused by bacterial infectious agents^59,60^; the indirect effects are often linked to genomic instability through direct genotoxic effects on DNA and by modulating epigenetic mechanisms^60^.

The development of breast cancer has been associated with intestinal microbiota dysbiosis since certain gut bacteria alter the production of the beneficial anticancer metabolites and disrupt estrogen metabolism^56^. There are plenty estrogen-dependent and non-estrogen-dependent functions of the gastrointestinal microbiome involved in the production of bioactive metabolites^56^. Also, regulation of free AAs across the course of its digestion and absorption is influenced by the resident gut microbiota and could reflect on the distribution in the gastrointestinal tract in mice^61^. Moreover, the gut microbiota performs a key function in producing AAs, and this includes de novo biosynthesis^61^. For instance, some AAs are essential for carbon skeletons such Leu, Trp, and His which cannot be synthetized by the cell and are required not only from the diet but also from the intestinal microbiota^62^. However, we reported, through our in vivo model, only alterations in the Leu levels. The role of AAs is also reported regarding the sulphate reduction that might result in genomic DNA damage. These AAs from the dietary protein contribute to H_2_S production by sulfate-reducing bacteria (SRB) and has been shown to have genotoxic effects in vitro^60^. Likewise, biogenic amines such as cadaverine were observed having an impact on the Trace amino acids receptors TAARs in mice with breast cancer^59^; in our study, we observed no alterations on the biogenic amines profile. Further investigation on how small molecules can affect cancer progression, particularly AAs, might be helpful in defining the functional relationship between microbiota and host. It is extremely necessary to classify AAs profile derived from tumor or the host physiology through metabolomics analysis based on liquid biopsies to increasingly correlate with their diurnal variation.

Caveats and limitations of the current study rely on the sections of experiments which were performed: i) We observed a not class-attributable variation between cancer cells (in vitro analysis) which were more spread compared to normal breast cells. The impairment regarding the replicability of the AAs profiling in cancer cells may be due to its normal variation which were also described by other studies. Our PLSDA models presented the lack of more accurate parameters (Supplementary figure 1); however, some metabolomics studies have reported a similar profile pattern in terms of more spread separation regarding cancer conditions. Past studies have already shown not only similar spread metabolite profile in cancer cells, but also metabolite variations observed in urine samples of cancer patients compared with healthy groups^63–65^. ii) The in vivo study limitation includes the use of a single animal model of tumors and is needed to confirm these findings in different human tumor xenograft models. Furthermore, although our data shows that AAs profile is not significantly altered by feed, the lack of a deeper investigation of the food intake habits may hinder the interpretation of AAs profile.

In summary, our report highlights the importance of the frequency of sampling and the potential effect of evaluating single or few time-points data which may not be appropriate in monitoring accurately tumor-related changes of circulating metabolites through daily rhythm. Our findings reveal an important variation of amino acids levels in MD-MB-231 breast cancer xenografts over the 24 h in contrast to a very low variation observed in isolated breast cancer cells. Therefore, additional experiments are needed to substantiate this effect in additional human tumor xenograft models of various genetic backgrounds. Although obvious advantages using human xenografts exist, the use of allograft syngeneic mouse models with reduced genetic and metabolic differences could generate valuable information to properly decipher the biological rhythms in cancer investigation from preclinical to clinical trials.

## Methods

### In vitro studies

#### Cell growth and culture methods

Human non-tumorigenic breast epithelial cell line MCF-10A (ATCC) was grown in 1:1 DMEM: Ham's F-12 (Life Technologies, Carlsbad, CA, EUA) media supplemented with 5% horse serum (Thermo Fisher Scientific, Waltham, MA, USA), epidermal growth factor (20 ng/mL) (Sigma-Aldrich, St. Louis, MO, USA), hydrocortisone (500 ng/mL) (Sigma-Aldrich, St. Louis, MO, USA), insulin (0.01 mg/mL) (Sigma-Aldrich, St. Louis, MO, USA) and cholera toxin (100 ng/mL) (Sigma-Aldrich, St. Louis, MO, USA). The triple negative breast cancer (TNBC) cell line MDA-MB-231 (ATCC, Manassas, VA, USA) was grown in 75 cm^2^ flasks (Sarstedt, Nümbrecht, Germany) with DMEM media (Life Technologies, Carlsbad, CA, EUA) supplemented with 10% fetal bovine serum (FBS) (Cultilab, Campinas, SP, Brazil), penicillin (100 U/mL) and streptomycin (100 mg/mL) (Sigma-Aldrich, St. Louis, MO, USA). Both cell lines were maintained in a humidified incubator at 5.0% CO2 at 37 °C until they were 80–90% confluent.

#### Intracellular metabolites extraction for targeted metabolomic analysis

Both cell lines were initially grown in complete media with an initial number of 2.1 × 10^6^ cells as described previously^22^. To analyze all classes of metabolites, 200 μL of sample extraction elution was performed with 50 nM of olomoucine (Sigma-Aldrich, St. Louis, MO, USA) in methanol (Greyhound Chromatography, Birkenhead, UK). After media was aspirated, cells were washed with PBS, and scraped into 200 µL cold methanol containing 50 nM of olomoucine. After 15 min on ice, the cells were centrifuged at ~ 2000 RCF for 30 min at 4 °C; 10 μL of the supernatants were used immediately for metabolomics analysis. The intracellular metabolites were normalized to the total protein using the bicinchoninic acid (BCA) protein assay kit according the manufacturer (Pierce BCA Protein Assay Kit), by dividing the AAs concentrations in the cells by the total protein absorbance in 200 µL of extraction solvent.

### In vivo experiments

#### Human breast cancer xenograft model

This study was given approval by the Ethics Committee on the Use of Animals of the Faculdade de Medicina de São José do Rio Preto FAMERP (Protocol 001–003,336 / 2014). The animal model and sampling were developed during 2014–2015 in accordance with the ARRIVE guidelines. All experimental procedures also followed the relevant guidelines and regulations according to national and international standards for ethics in animal experimentation. A total of 80 Balb/c nu female mice, 7–8 weeks of age, weighing 16–18 g were purchased from the Central Biotherium of the FMUSP (São Paulo, Brazil). Animals were kept in pathogen-free conditions, at 21 to 25 °C and exposed to a normal diurnal variation under 12 h of light and 12 h of dark with food and water available ad libitum.

MDA-MB-231 human tumor breast cells were grown, harvested and re-suspended with serum free media at a concentration of 6 × 10^7^ cells per mL. Mice were subcutaneously inoculated with 100 µL of cell suspension in the fat pad, which requires more time for consistent growth compared with hind flank^22–25^, in addition to accounting for reduced tumor volumes. The animals were randomly divided into two groups: non-tumor bearing (n = 5) and tumor bearing (n = 5), which were partitioned into 8 time points within 24 h. The control group received 100µL of vehicle solution to subject the animals to the same stress handling. Mice were periodically weighed and monitored during the experimental period and showed no variation within the groups. Tumor volumes were measured by sample, at each time point by digital caliper and calculated based on the measurements by the formula: tumor volume = 0.5 (length × width^2^).

#### Plasma sampling for targeted metabolomic analysis

Mice were anesthetized with ketamine–xylazine combination and euthanized by open cardiac puncture at eight time points (Zeitgeber time—ZT) every three hours over a 24 h period: 06:00 am (ZT0), 09:00 am (ZT3), 12:00 pm (ZT6), 03:00 pm (ZT9), 06:00 pm (ZT12), 09:00 pm (ZT15); 12:00 am (ZT18) and 03:00 am (ZT21). During the dark time points, the sampling was performed under systematic and standardized steps. We avoided artificial light inside and near the vivarium, by using red dim light to illuminate the room whilst blood sampling was carried out on the bench. The blood was collected using a heparinized syringe and the plasma was subsequently extracted by centrifugation at ~ 5,000 RCF for 2 min at 4 °C. The supernatant was recovered and stored at—80 °C until further analysis. Mice were not fasted, not only because of the number of animals but also due to the number of time points. The sampling frequency of most chronobiology studies is typically 4–6 h and we achieved more time-points and collection over a longer duration. Therefore, it would be rather difficult to coordinate the sampling with the fasting conditions as well.

#### Targeted metabolomic analysis

Metabolites were measured using the AbsoluteIDQ p180 targeted metabolomics kit (Biocrates Life Sciences AG, Innsbruck, Austria) which covers 6 classes of metabolites including amino acids, biogenic amines, acylcarnitines, phosphatidylcholines, lysophosphatidylcholines, and sphingomyelins, on a Waters Xevo TQ-S mass spectrometer coupled to an Acquity H-Class LC system (Waters Corporation, Milford, MA, USA).

### Data analysis

Data were processed with MassLynx V4.1 and validated by MetIDQ software (Biocrates Life Sciences AG, Innsbruck, Austria). The first analysis were performed through Principal Component Analysis (PCA) followed by Partial Last Squares Discriminant Analysis (PLS-DA) by Metabonalyst 5.0. In sequence, heatmaps analysis per each class of metabolite were also performed through Metabonalyst 5.0 (Supplementary figures). The p values (< 0.05) used were generated from the rhythmic analysis for every single AAs between the groups in each time points for both assays (in vitro and in vivo); two-way analysis of variance adjusted by Bonferroni test was performed using GraphPad Prism. For the enrichment pathway evaluation we still used Metaboanalyst 5.0 database. The heatmaps used for clustering analyses (Fig. 3) were performed using the web tool Morpheus (https://software.broadinstitute.org/morpheus).

### Ethics approval and guidelines

The study was performed in accordance with the ten standard items for ethics in animal experimentation described on the ARRIVE guidelines. The project was approved by the Ethics Committee on the Use of Animals of the Faculdade de Medicina de São Jose do Rio Preto—FAMERP (Protocol 001-003336 / 2014).

## Supplementary Information

Supplementary Figure S1.

Supplementary Figure S2.

Supplementary Legends.

## Abbreviations

AAs: Amino acids
ANOVA: Analysis of variance
ASS1: Argininosuccinate Synthetase 1
BCA: Bicinchoninic Acid
BCAA: Branched-chain Amino Acids
BMAL1: Brain and Muscle ARNT-Like 1
CLOCK: Circadian Locomotor Output Cycles Kaput
C_max_: Maximum plasma concentration
C_min_: Minimum plasma concentration
DMEM: Dulbecco’s Modified Eagle’s Medium
FBS: Fetal Bovine Serum
GSA: Glutamic-c-semialdehyde
Lyso PC: Lysophosphatidylcholine
MCF-10A: Michigan Cancer Foundation-10A
MCF-7: Michigan Cancer Foundation-7
MDA-MB-231: MD Anderson Metastatic Breast-231
NADPH: Nicotinamide Adenine Dinucleotide Phosphate
OTC: Ornithine Transcarbamylase
PBS: Phosphate Buffered Saline
Per2: Period Circadian Regulator 2
PFAA: Plasma Free Amino Acids
PLSDA: Partial Least-Squares Discriminant Analysis
PRODH: Proline Dehydrogenase
RCF: Relative Centrifugal Force
RORγ: RAR-related Orphan Receptor Gamma
SRB: Sulfate-reducing Bacteria
TAARs: Trace Amino acids Receptors
TQ-S: Triple Quadrupole Spectrometer
ZT: Zeitgeber time
