# Supplementary figures and images for: Presence of human breast cancer xenograft changes the diurnal profile of amino acids in mice
Source: Sci Rep. 2022 Jan 19;12:1008. doi: 10.1038/s41598-022-04994-6 (PMC8770691; doi:10.1038/s41598-022-04994-6)

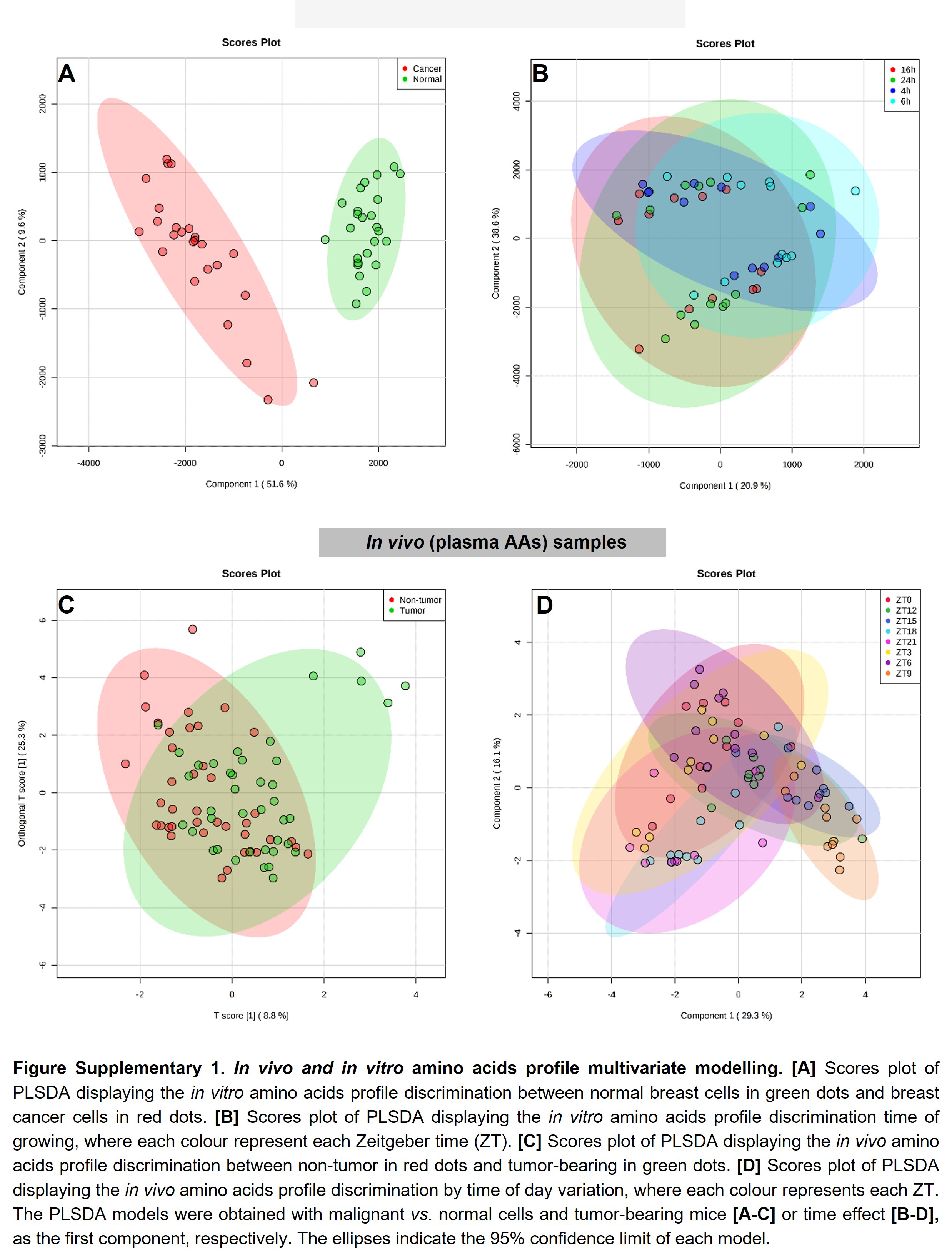

Supplement: Supplementary file 1 — Supplementary Figure S1. [file 41598_2022_4994_MOESM1_ESM.tif]

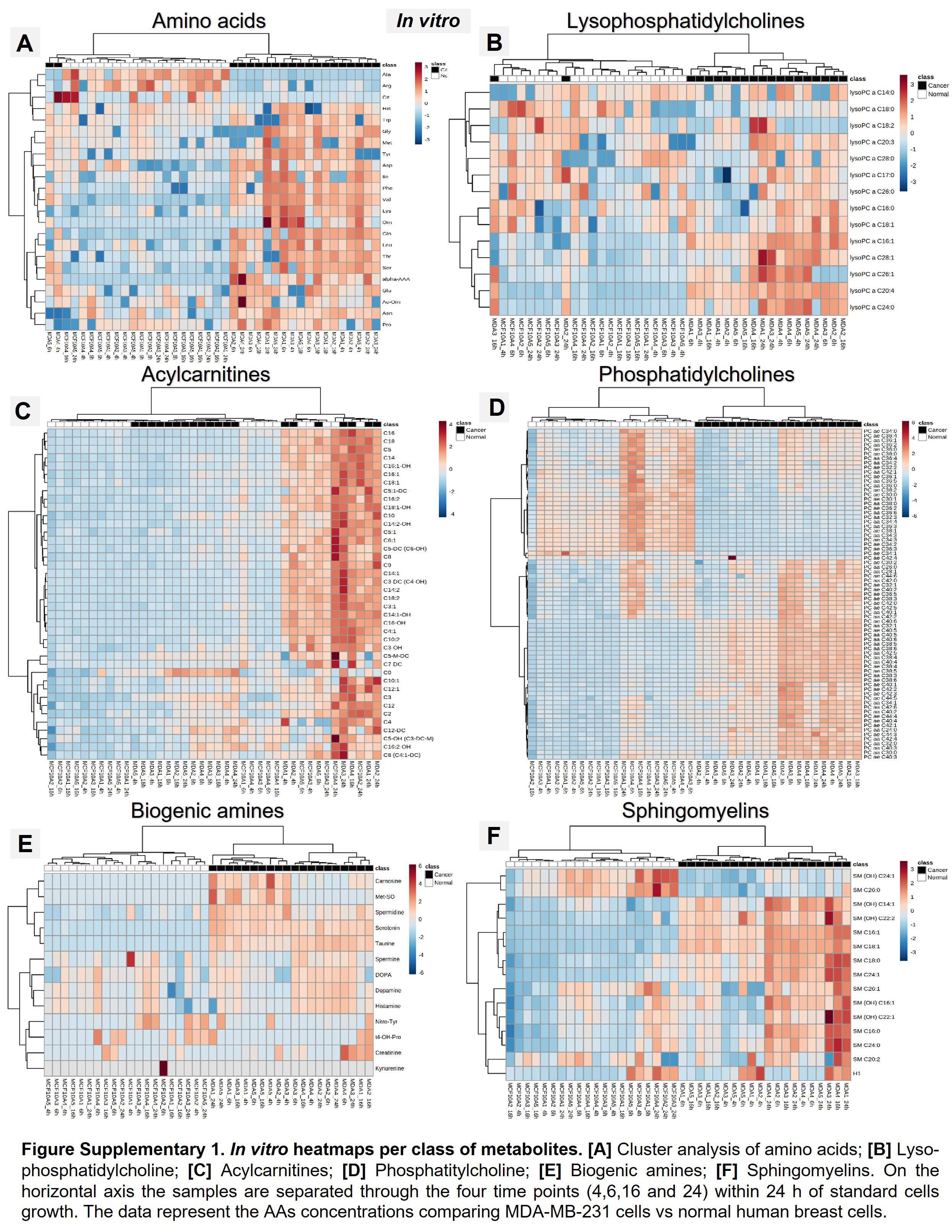

Supplement: Supplementary file 2 — Supplementary Figure S2. [file 41598_2022_4994_MOESM2_ESM.tif]
