# Supplementary material for: Presence of human breast cancer xenograft changes the diurnal profile of amino acids in mice
Source: Sci Rep. 2022 Jan 19;12:1008. doi: 10.1038/s41598-022-04994-6 (PMC8770691; doi:10.1038/s41598-022-04994-6)
Supplement: Supplementary file 3 — Supplementary Legends. [file 41598_2022_4994_MOESM3_ESM.docx]

**Supplementary Material**

**Figure Supplementary 1. *In vivo and in vitro* amino acids profile multivariate modelling.** **[A]** Scores plot of PLSDA displaying the *in vitro* amino acids profile discrimination between normal breast cells in green dots and breast cancer cells in red dots. **[B]** Scores plot of PLSDA displaying the *in vitro* amino acids profile discrimination time of growing, where each colour represent each Zeitgeber time (ZT). **[C]** Scores plot of PLSDA displaying the *in vivo* amino acids profile discrimination between non-tumor in red dots and tumor-bearing in green dots. **[D]** Scores plot of PLSDA displaying the *in vivo* amino acids profile discrimination by time of day variation, where each colour represents each ZT. The PLSDA models were obtained with malignant *vs.* normal cells and tumor-bearing mice **[A-C]** or time effect **[B-D],** as the first component, respectively. The ellipses indicate the 95% confidence limit of each model.

**Figure Supplementary 2. In vitro heatmaps per class of metabolites. [A]** Cluster analysis of amino acids; **[B]** Lyso-phosphatidylcholine; **[C]** Acylcarnitines; **[D]** Phosphatitylcholine; **[E]** Biogenic amines; **[F]** Sphingomyelins. On the horizontal axis the samples are separated through the four time points (4,6,16 and 24) within 24 h of standard cells growth. The data represent the AAs concentrations comparing MDA-MB-231 cells vs normal human breast cells.
